# HEK293 producing the extracellular domain HER1: Full datasets of continuous fermentation process and metabolites analysis

**DOI:** 10.1016/j.dib.2023.109604

**Published:** 2023-09-21

**Authors:** Lisandra Calzadilla, Erick Hernández, Julio Dustet, Jorge Fernandez-de-Cossio-Diaz, Kalet León, Matthias Pietzke, Alexei Vazquez, Roberto Mulet, Tammy Boggiano

**Affiliations:** a Center of Molecular Immunology, Bioprocesses Development Division. 216 Street and 15 Avenue, Atabey, HavanaX, Cuba; b Technological University of Havana José Antonio Echeverría, Chemical Engineering Faculty. 114 Street, No. 11901, Marianao, Havana PO 11600, Cuba; c Laboratory of Physics of the Ecole Normale Supérieure, CNRS UMR 8023 & PSL Research, Sorbonne Université, 24 rue Lhomond, 75005 Paris, France; d Cancer Research UK Beatson Institute, Garscube State, Switchback Road, Bearsden PO G61 1BD, Glasgow, UK; e Institute of Cancer Sciences, University of Glasgow, Glasgow PO G61 1QH, UK; f University of Havana, Physics Faculty. San Lázaro and L street, Plaza de la Revolución, Havana PO 10400, Cuba

**Keywords:** HEK293 cell line, Continuous culture dataset, Metabolic dataset, PCA analysis

## Abstract

The data for provide evidences of the multi steady state of the human cell line HEK 293 was obtained from 2 L bioreactor continuous culture. A HEK 293 cell line transfected to produce soluble HER1 receptor was used. The bioreactor was operated at three different dilution rates in sequential manner. Daily samples of culture broth were collected, a total of 85 samples were processed. Viable cell concentration and culture viability was addressing by trypan blue exclusion method using a hemocytometer. Heterologous HER1 supernatant concentration was quantified by a specific ELISA and the metabolites by mass spectrometry coupled to a liquid chromatography.

The primary data were collected in excel files, where it was calculated the kinetic and other variables by using mass balance and mathematical principles. It was compared the steady states behavior each other's to find out the existence of steady states’ multiplicity, taking into account the stationary phase with respect to the cell density (which means its coefficient of variation is less than 20 %).

From the metabolic measurements by using Liquid Chromatography coupled to mass spectrometry (LC-MS), it was also built the data matrix with the specific rates of the 76 metabolites obtained. The data were processed and analyzed, using multivariate data asssnalysis (MVDA) to reduce the complexity and to find the main patterns present in the data.

We describe also the full data of the metabolites not only for steady states but also in the time evolution, which could help others in terms of modeling and deep understanding of HEK293 metabolism, especially under different culture conditions.

Specifications Table

**Table:** 

Subject	Biological science
Specific subject area	Cell culture biotechnology and metabolic profiling
Type of data	Table Figure
How the data were acquired	The data were acquired from different sources listed below: Viable cell density and viability, via optical microscope by trypan blue dye exclusion method. The data were recorded at the electronic notebook. Extracellular domain HER1 concentration, via microplate photometer by ELISA sandwich method (GEN5 Reader Control Software). Metabolites concentration, via Liquid Chromatography-Mass spectrometry (LC-MS), where metabolites are first separated by their retention time and later by their specific mass/charge (*m/z*).
Data format	Raw Analyzed Filtered
Description of data collection	The data correspond to the fermentation process in continuous mode of HEK293 cells producing the heterologous glycoprotein HER1, in a 2 L bioreactor. Samples were taken daily from the culture for subsequent analysis of cell concentration, protein concentration and metabolites measurement. The metabolic data were autoscaling-standardized (ratio of centered mean and the standard deviation) prior the analysis*.*
Data source location	Cells and protein concentrations: · Institution: National Institute of Molecular Immunology. Center of Molecular Immunology · City/Town/Region: La Habana · Country: Cuba · Latitude and longitude (and GPS coordinates, if possible) for collected samples/data: https://goo.gl/maps/vu2MfxXzTHRywLLD7 Metabolite measurement: · Institution: Beatson Institute for Cancer Research · City/Town/Region: Glasgow · Country: UK · Latitude and longitude (and GPS coordinates, if possible) for collected samples/data: https://goo.gl/maps/1ufVnD2sAXBTEnAN8
Data accessibility	Pre-processed data is available at Mendeley Data [1] Data identification number: 10.17632/t9rcjv5362.2 Direct URL to data: https://data.mendeley.com/datasets/t9rcjv5362/2

## 1 Value of the Data

• The data we present correspond to the continuous culture of a human cell line: HEK293. It is presented not only the cell density and heterologous protein evolution across the whole culture time, but also the metabolic behavior of 76 metabolites for each point. These results give us the opportunity to carry out a very exhaustive study in order to characterize the metabolism and find the main patterns in this cell line over this growing regime.
• The study of the phenomenon of multiplicity of steady states in this cell line is one of the main goals of our work. The data describe this phenomenon. We think that this could be of interest for researchers linked not only in basic research but also for those associated to the research and development step, considering that is experimentally tested a new strategy to stimulate metabolic changes in this cell line. Also, the community of theoretic science will find this information very useful.
• The data can be used with modeling purpose for the understanding of the plasticity of HEK293 metabolism, specially, under different metabolic patterns.

### 1.1 Objective

The comprehension of metabolism is a key target in Bioprocesses development optimization and scaling up processes. The current paper describes the full collected data from the cell culture of HEK293 cell line producing the extracellular domain HER1 in a 2 L bioreactor. The cell culture was carried out in continuous mode, and the stimulation to trigger a metabolic switch was done, reaching several steady states, and some of those coexist at the same dilution rate. The data includes not only the standard cell culture variables, but also the data of a wide number of metabolites (76 metabolites across the culture time of 86 days), that there were quantified by Liquid Chromatography-Mass spectrometry analytical technique.

## 2 Data Description

HEK293 cell line, producing the extracellular domain HER1 (ECD-HER1), was cultured in continuous mode using a 2 L bioreactor for 86 days. It was experimentally assayed different dilution rates (D) in order to obtain several steady states. Table 1 describes the data generated from the daily culture sampling, such as the culture time, the cell density, the ECD-HER1 concentration, the corresponding D, the zone where the steady states were defined, and other variables. The full information of the rest of variables associated to the culture can be found at the repository as an excel file (.xlsx) named as **2** **L continuous culture-Extracellular domain HER1**.

**Table 1:** Cell culture variables across the culture time.

Steady state	Sample	Runtime	Xv	D	Protein Titer	qP
Units	#	h	Cells/mL	(1/d)	µg/mL	(PCD)
	1	0,48	4,63E + 05	0,00	1,70	
	2	23,76	8,50E + 05	0,00	4,52	4,43
	3	46,8	1,75E + 06	0,00	11,36	5,47
	4	69,6	2,14E + 06	0,30	12,87	2,69
SS1	5	93,6	3,27E + 06	0,44	14,37	2,78
SS1	6	120,72	2,82E + 06	0,46	14,37	2,18
SS1	7	149,28	2,66E + 06	0,44	14,32	2,30
SS1	8	167,28	2,20E + 06	0,44	14,27	2,54
SS1	9	191,76	2,81E + 06	0,46	15,13	3,05
SS1	10	214,08	2,90E + 06	0,46	16,65	3,13
SS1	11	237,84	2,88E + 06	0,45	16,87	2,71
SS1	12	261,6	3,21E + 06	0,46	16,04	2,19
SS1	13	293,04	2,87E + 06	0,44		
	14	312,48	2,38E + 06	0,40	9,57	5,97
	15	361,2	3,35E + 06	0,41	1,28	
	16	382,56	3,67E + 06	0,39	1,24	0,13
	17	405,6	4,25E + 06	0,39	1,20	0,11
	18	457,44	5,19E + 06	0,40	0,93	0,06
	19	479,52	6,38E + 06	0,41	2,65	0,45
	20	509,52	7,25E + 06	0,40	3,01	0,21
	21	531,6	6,36E + 06	0,41	3,14	0,20
	22	552,48	6,90E + 06	0,40	3,26	0,21
	23	573,84	6,85E + 06	0,40	3,44	0,22
SS2	24	598,32	8,00E + 06	0,41		
SS2	25	629,76	7,47E + 06	0,40	11,84	
SS2	26	648,72	8,28E + 06	0,41	11,14	0,49
SS2	27	675,6	8,83E + 06	0,40	11,30	0,55
SS2	28	700,8	8,76E + 06	0,40	9,94	0,34
SS2	29	720,96	8,81E + 06	0,39	11,46	0,68
SS2	30	795,6	8,80E + 06	0,35	12,15	0,50
	31	844,56	9,71E + 06	0,35	19,83	1,02
	32	870,48	9,38E + 06	0,35	24,85	1,31
	33	909,6	7,49E + 06	0,35	31,51	1,66
	34	933,6	6,90E + 06	0,35	32,18	1,64
SS3	35	988,56	5,48E + 06	0,35	23,87	1,00
SS3	36	1038,24	5,60E + 06	0,35	18,78	0,90
SS3	37	1054,8	5,31E + 06	0,36	17,93	1,00
SS3	38	1077,6	5,40E + 06	0,34	14,53	
SS3	39	1132,08	5,33E + 06	0,35	12,07	0,67
SS3	40	1150,56	5,61E + 06	0,36		
SS3	41	1175,76	5,19E + 06	0,41	8,91	2,24
	42	1229,52	5,17E + 06	0,40	9,71	0,79
	43	1299,36	6,11E + 06	0,40	12,12	0,93
	44	1324,32	5,59E + 06	0,40	9,64	0,33
	45	1345,2	6,09E + 06	0,39	10,91	0,93
	46	1391,52	6,75E + 06	0,40	9,03	0,47
	47	1416	7,33E + 06	0,41	8,47	0,44
	48	1487,04	7,94E + 06	0,40	7,91	0,40
	49	1511,52	9,21E + 06	0,39	9,14	0,53
	50	1536,72	7,47E + 06	0,40	10,37	0,61
SS4	51	1560,72	9,33E + 06	0,40	12,60	0,81
SS4	52	1631,28	1,08E + 07	0,40	14,16	0,59
SS4	53	1657,2	1,05E + 07	0,39	20,08	1,15
SS4	54	1684,56	8,63E + 06	0,40	13,04	
SS4	55	1709,28	9,60E + 06	0,39	15,16	0,83
SS4	56	1726,8	9,75E + 06	0,41	17,26	0,98
SS4	57	1749,6	9,17E + 06	0,40	16,50	0,62
SS4	58	1852,8	1,08E + 07	0,40	19,93	0,81
SS4	59	1876,8	1,07E + 07	0,40	18,22	0,55
SS4	60	1895,28	1,07E + 07	0,39	26,36	
SS5	61	1967,52	1,04E + 07	0,45	23,28	0,96
SS5	62	1996,8	1,03E + 07	0,45	25,00	1,19
SS5	63	2021,28	9,77E + 06	0,44	23,51	0,92
SS5	64	2046,48	1,10E + 07	0,45	24,42	1,12

**Fig. 1:**
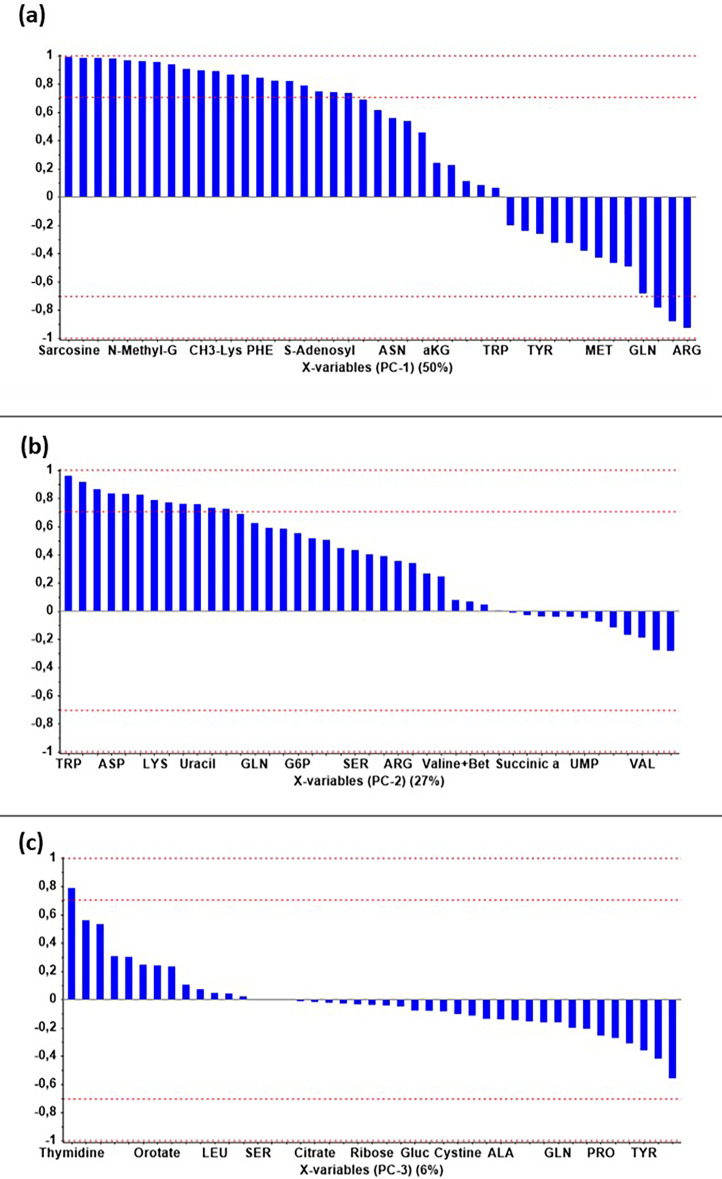
Correlation loadings of metabolites on the corresponding Principal Component (PC). It is taken into consideration ± 0,7 as the significant correlation limit. Panel (a): PC1 representation. Panel (b): PC2 representation. Panel (c): PC3 representation. Principal Component Analysis (PCA) results for metabolites specific uptake and production rates´ matrix. The correlation loading results are presented below.

On the other hand, from the daily samples, the measurement of 76 metabolites was carried out by using Liquid Chromatography-Mass Spectrometry (LC-MS), and Supplementary table T1 collects the name of those measured metabolites. The specific rates of metabolites were calculated from the mass balance equations [2,3], having the metabolites output measurement and the medium formulation. We create a data matrix, with the specific uptake or production rates of the metabolites and the steady states, organized in a variable wise mode, which can be found also at the repository as excel files (.xlsx) named as qS Matrix_Area. Multivariate data analysis (MVDA) approaches, specifically Principal Component Analysis (PCA), was spread over the matrix mentioned above, in order to find the main patterns in the data. With the aim of finding the correspondence of each metabolite with the main patterns in the data, we also compute the correlation loading graph, and Fig. 1 show the results. Full information of MVDA results can be found at the repository files, like the standardization of the data, the selection of significant principal component (PC), the correlation loading for each PC, and the grouping of metabolites depending on their corresponding PC. Specifically, the MVDA results are saved as Unscrambler files (.unsb), named as .

## 3 Experimental Design, Materials and Methods

### 3.1 Cell culture protocol

**Figure:**
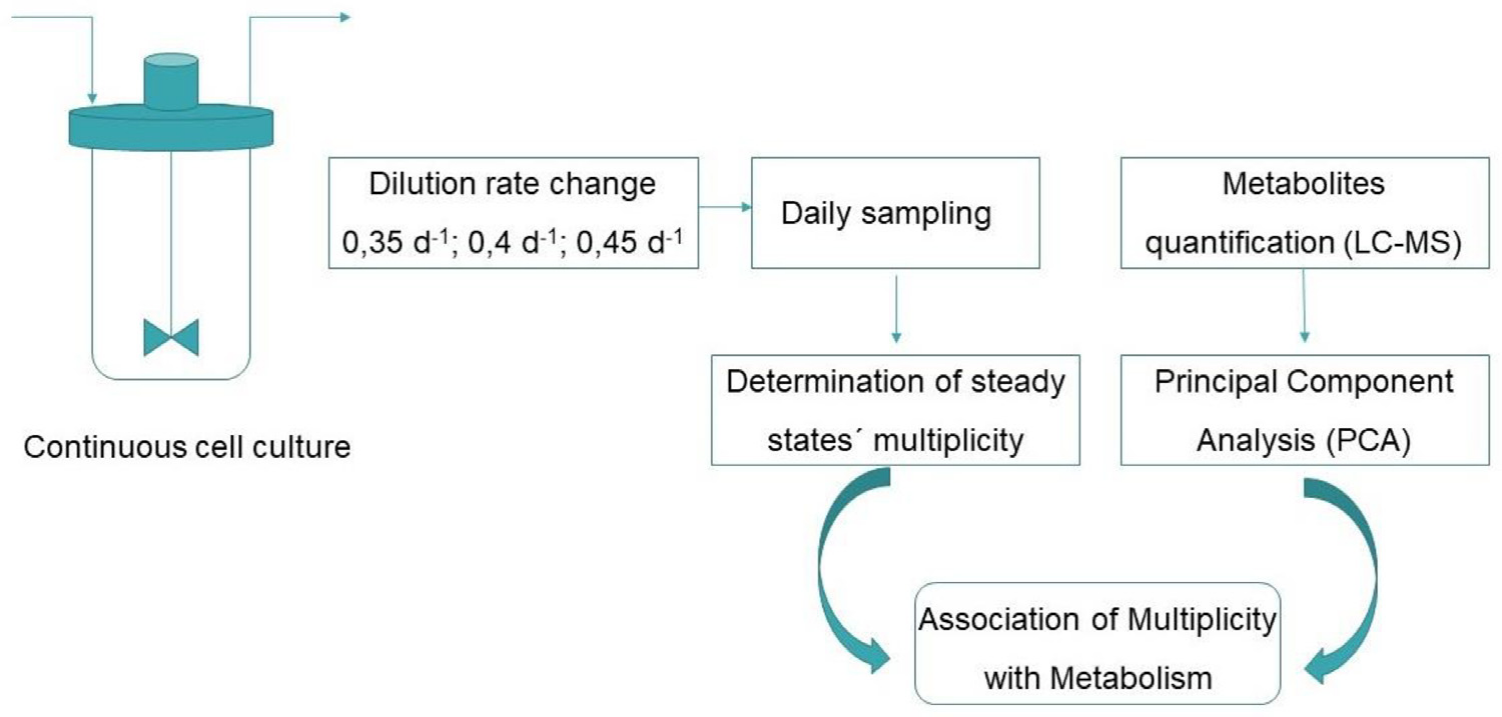

In order to test the multiplicity of steady states in HEK293 cell line, cells were culture in continuous mode, keeping the same medium formulation (which can be found at the repository). Three D (0,35 d^−1^; 0,4 d^−1^ and 0,45 d^−1^) were assayed. The continuous operation was started at 0,45 d^−1^ followed by 0,4 d^−1^ and later was reduced down to 0,35 d^−1^. Then, D was progressively increased to 0,4 d^−1^ and later 0,45 d^−1^ once more. Each steady state was kept for at least four days after the stabilization (which means coefficient of variation of cell density less than 20 %).

Samples were taken from the culture on daily base to quantify cell concentration, viability, ECD-HER1 concentration and metabolites concentration.

### 3.2 Quantification methods and data analysis

Cell density and viability were determined by trypan blue dye exclusion method in Neubauer chamber and using optical microscopy. It was applied the sample dilution in trypan blue depending on the expected cell density.

On the other side, the ECD-HER1 concentration was determined by a homemade ELISA sandwich method. 96-microwell plates were coated with 5 mg/mL of an anti-HER1 (Center of Molecular Immunology propriety), at least the day prior to the assay and kept at 4 °C. At the moment of the assay, samples and standard are bringing to room temperature, and serial dilutions are applied to them in order to obtain the samples concentration in the curve range. Then, they are added to the plates and incubated at 37 °C 1 h. Three automated washing step is applied, and then an anti-EGFr antibody conjugated with biotin (R&D system) is added to the plate and incubated at 37 °C 1 h. Three automated washing step is applied again, and Streptavidin-Peroxidase reagent (R&D system) is added to the plate and incubated at 37 °C 1 h. The plates are automated washed for three times. Finally, TMB substrate (R&D system) is added to the plate. After 20 min the reaction is stopped with sulfuric acid and the plates are read by a spectrophotometer at 450 nm.

On the other hand, the metabolites measurement was carried out by LC-MS technique. Metabolites are primarily extracted from the samples, that are diluted 1:100 in extraction solvent, LC-MS grade (ACN:MeOH:H2O, 3:5:2) [4]. Then, diluted samples are well mixed in vortex for 20 s and centrifuged at 14,000 g x 10 min and 4 °C. At that point, samples are transferred to LC-MS vials for separation and detection in the LC-MS equipment. Metabolites are separated by the LC column ZIC-pHILIC column (Merck Millipore) with depend of their retention times, using metabolites’ standards from Sigma Aldrich (Merck Millipore). For the separation it is used 20 mM ammonium carbonate as aqueous mobile phase solvent, adjusted to pH 9.4 with 0.1 % ammonium hydroxide solution (25 %); and 100 % Acetonitrile as organic mobile phase. It is applied a lineal gradient strategy at 200 mL/min for the separation process that take around 15 min, and followed by an equilibration step. Column is kept in the oven at 45 °C, and samples are maintained at 4 °C prior to injection to the Q-Exactive mass spectrometer with auto-sampler, Heater Electro Spray Ionization source (HESI), and ORBITRAP as detector (AGILENT). Finally, the metabolites are separated by their mass/charge (*m/z*) with a mass accuracy below to 5 ppm with the Q-Exactive mass spectrometer. The LC-MS raw data is then preprocessed using TraceFinder software.Finally, the MVDA was used to analyze the metabolic data, considering its application for improving the data analysis with a large number of variables. We specifically used PCA method. The MVDA was carried out by using The UNSCRAMBLER X (version 10.4, Camo Software). The data was standardized with autoscaling method, using the equation presented below:

$${\tilde{x}}_{ij}=\frac{x_{ij}-{\bar{x}}_{i}}{s_{i}}$$

## Ethics Statements

This work does not include data from human subjects, animal experiments or data collected from social media platforms.

## CRediT authorship contribution statement

**Lisandra Calzadilla:** Methodology, Investigation, Formal analysis, Data curation, Writing – original draft, Visualization. **Erick Hernández:** Formal analysis, Data curation. **Julio Dustet:** Supervision. **Jorge Fernandez-de-Cossio-Diaz:** Conceptualization. **Kalet León:** Conceptualization. **Matthias Pietzke:** Resources. **Alexei Vazquez:** Resources, Supervision, Funding acquisition. **Roberto Mulet:** Conceptualization, Methodology, Supervision, Writing – review & editing. **Tammy Boggiano:** Conceptualization, Methodology, Supervision, Writing – review & editing.

## Data Availability

Continuous culture of HEK293 producing the ECD-Her1. Existence steady states' multiplicity at the same external conditions.Cell culture and metabolic datasets (Original data) (Mendeley Data).

## Appendix Supplementary Materials

Image, application 1
