# Supplementary material for: HEK293 producing the extracellular domain HER1: Full datasets of continuous fermentation process and metabolites analysis
Source: Data Brief. 2023 Sep 21;50:109604. doi: 10.1016/j.dib.2023.109604 (PMC10556581; doi:10.1016/j.dib.2023.109604)
Supplement: Supplementary file 1 [file mmc1.docx]

Table 2_Metabolites quantified by LC-MS and used for multivariate analysis of steady states in continuous mode.

| Butyric acid | Cystathionine | Glucose | Pyridoxal |
| --- | --- | --- | --- |
| Citric acid | Cysteate | Glucose-6-phosphate | Riboflavin |
| Decanoic acid | Cys-Gly | Glutamate | Ribose |
| Stearic acid | Cystine | Glutamine | Phenol red |
| Hexanoic acid | Cytidine | Hypoxanthine | S-Adenosyl-L-Homocysteine |
| Linoleic acid | Cytosine monophosphate (CMP) | Isoleucine | Sarcosine |
| Miristic acid | Citrulline | Lactate | Serine |
| Oleic acid | Dihydroorotate | Leucine | Succinate |
| Palmitic acid | Dihydroxyacetone phosphate (DHAP) | Lysine | Succinyl-adenosine |
| Palmitoleic acid | Spinacine | Malate | Succinyl-cysteine |
| Adenine | Phenylalanine | N-Methyl-Glutamate | Thymidine |
| aKG | Folate | Methyl-Lysine | Tyrosine |
| Alanine | Fosfatidil-colina | Methionine | Threonine |
| Arginine | Phosphoenolpyruvate | Nicotinamide | Tryptophan |
| Arginosuccinate | Ethanolamine phosphate | Ornithine | Uracil |
| Asparagine | Fumarate | Orotate | Uridine diphosphate-N acetyl glucosamine  GlcNAc-UDP |
| Aspartato | Glyceraldehyde 3-phosphate | Oxypurinol | Uridine monophosphate |
| Carnitine | Glycerol 3- phosphate | Pyruvate | Valine |
| Cis-Aconitate | Glycine | Proline | Valine Betaine |
